# The role of co-occurring conditions and genetics in the associations of eating disorders with attention-deficit/hyperactivity disorder and autism spectrum disorder

**DOI:** 10.1038/s41380-024-02825-w

**Published:** 2024-11-14

**Authors:** Gitte Bundgaard Christiansen, Liselotte Vogdrup Petersen, Hannah Chatwin, Zeynep Yilmaz, Diana Schendel, Cynthia M. Bulik, Jakob Grove, Isabell Brikell, Birgitte Dige Semark, Katrine Holde, Mohamed Abdulkadir, Christopher Hübel, Clara Albiñana, Bjarni Jóhann Vilhjálmsson, Anders D. Børglum, Ditte Demontis, Preben Bo Mortensen, Janne Tidselbak Larsen

**Affiliations:** 1 The National Centre for Register-based Research, Aarhus BSS, Aarhus University, Aarhus, Denmark; 2 The Lundbeck Foundation Initiative for Integrative Psychiatric Research (iPSYCH), Aarhus, Denmark; 3 Department of Biomedicine, Aarhus University, Aarhus, Denmark; 4 Department of Medical Epidemiology and Biostatistics, Karolinska Institutet, Stockholm, Sweden; 5 Department of Psychiatry, School of Medicine, University of North Carolina at Chapel Hill, Chapel Hill, NC USA; 6 AJ Drexel Autism Institute, Drexel University, Philadelphia, PA USA; 7 Department of Nutrition, University of North Carolina at Chapel Hill, Chapel Hill, NC USA; 8 Center for Genomics and Personalized Medicine, Aarhus, Denmark; 9 Bioinformatics Research Center, Aarhus University, Aarhus, Denmark; 10 Social, Genetic & Developmental Psychiatry Centre, Institute of Psychiatry, Psychology & Neuroscience, King’s College London, London, UK; 11 Department of Pediatric Neurology, Charité – Universitätsmedizin Berlin, Berlin, Germany; 12 The Novo Nordisk Foundation Center for Genomic Mechanisms of Disease, Broad Institute of MIT and Harvard, Cambridge, MA USA; 13 The Centre for Integrated Register-based Research at Aarhus University (CIRRAU), Aarhus, Denmark

**Keywords:** Psychiatric disorders, Genetics

## Abstract

Eating disorders (EDs) commonly co-occur with other psychiatric and neurodevelopmental disorders including attention-deficit/hyperactivity disorder (ADHD) and autism spectrum disorder (ASD); however, the pattern of family history and genetic overlap among them requires clarification. This study investigated the diagnostic, familial, and genetic associations of EDs with ADHD and ASD. The nationwide population-based cohort study included all individuals born in Denmark, 1981–2008, linked to their siblings and cousins. Cox regression was used to estimate associations between EDs and ADHD or ASD, and mediation analysis was used to assess the effects of intermediate mood or anxiety disorders. Polygenic scores (PGSs) were used to investigate the genetic association between anorexia nervosa (AN) and ADHD or ASD. Significantly increased risk for any ED was observed following an ADHD or ASD diagnosis. Mediation analysis suggested that intermediate mood or anxiety disorders could account for 44%–100% of the association between ADHD or ASD and ED. Individuals with a full sibling or maternal half sibling with ASD had increased risk of AN compared to those with siblings without ASD. A positive association was found between ASD-PGS and AN risk whereas a negative association was found between AN-PGS and ADHD. In this study, positive phenotypic associations between EDs and ADHD or ASD, mediation by mood or anxiety disorder, and genetic associations between ASD-PGS and AN and between AN-PGS and ADHD were observed. These findings could guide future research in the development of new treatments that can mitigate the development of EDs among individuals with ADHD or ASD.

## Introduction

Eating disorders (EDs), including anorexia nervosa (AN), bulimia nervosa (BN), and eating disorder not otherwise specified (EDNOS), are severe psychiatric disorders associated with impaired quality of life and high comorbidity and relative mortality [1]. EDs co-occur with neurodevelopmental conditions including attention-deficit/hyperactivity disorder (ADHD) and autism spectrum disorder (ASD) [2], as well as mood—including major depressive and bipolar disorder—and anxiety disorders [3]. The lifetime prevalence of ADHD in EDs has been reported to range between 1.6% and 18%, while the lifetime prevalence of ASD in EDs has been reported to be on average 4.7% [2]. Mood and anxiety disorders have been reported to be the most prevalent psychiatric comorbidities in individuals with EDs [3, 4] with comorbidity rates of up to 54% and 62%, respectively [4]. Individuals with EDs and co-occurring ADHD or ASD often experience more severe ED symptoms, need longer time to recover, and have a longer duration of inpatient treatment than individuals with EDs without co-occurring neurodevelopmental conditions [5–7].

The co-occurrence of EDs with ADHD and ASD suggests potential etiologic overlap across these disorders. Familial and genetic studies have investigated the source of the overlap, and results have been mixed. Twin studies have reported low cross-twin, cross-trait correlations between eating problems and ADHD and/or ASD [8], and a twin-based genetic correlation of 0.35 between current ADHD symptoms and lifetime binge-eating behavior was observed [9]. A Swedish study reported familial co-aggregation and a genetic association between ADHD and EDs, which was weaker for AN than for other EDs [10]. In recent large genome-wide association studies (GWASs) for ADHD [11, 12], ASD [13], and AN [14], risk loci have been identified for all three disorders. Despite comorbidity between AN and ADHD, a significant negative [11] or no [12] genetic correlation after Bonferroni correction was reported while no significant genetic correlation after Bonferroni correction was found between AN and ASD [13, 14]. An ADHD polygenic score (PGS) showed a significant positive association with binge-eating disorder, and an ASD-PGS was negatively associated with AN, although this finding was not significant [15]. Frequent co-occurrence of mood and anxiety disorders with EDs [16, 17], ADHD, and ASD also suggest potential etiologic overlap, but to our knowledge, no studies have examined the role of intermediate mood or anxiety disorders on ED–ADHD–ASD associations.

To address these knowledge gaps, we conducted a Danish register-based cohort study of patterns of diagnostic co-occurrence between EDs and ADHD or ASD, including an exploration of the role of intermediate mood or anxiety disorders on the diagnostic patterns, and shared familial and genetic factors.

## Materials and methods

### Study population

The study population comprised all individuals born in Denmark between May 1, 1981, and December 31, 2008, who were alive and residing in Denmark on their sixth birthday (*N* = 1,690,087). Individuals whose parents could not be identified in the Danish Civil Registration System [18] (CRS; *N* = 11,512) or who were adopted (*N* = 6752) were excluded, resulting in a final sample of 1,671,823 individuals. We used data from the CRS to link these individuals to their full siblings, maternal and paternal half siblings, and cousins within the study population. We restricted family linkage to individuals within the study population so that the same diagnostic system (International Classifications of Disease-Tenth Edition, ICD-10) and time frame for diagnosis (1994 and onwards) applied to all family members. Information on diagnoses before 1994 was not included. Consequently, some cases were not captured, and some cases are not incidence. Linkage was achieved via the unique personal identification number assigned to all Danish citizens.

### Outcome and covariate data

Each cohort member and relative was followed until December 31, 2016, for a reported ICD-10 diagnosis of AN, BN, EDNOS, any ED, ADHD, ASD, mood disorder, or anxiety disorder as described in Table 1. Individuals were considered being at risk of ADHD and ASD from birth and EDs, mood, and anxiety disorders from age 6. The EDNOS case group includes EDs such as binge-eating disorder, avoidant/restrictive food intake disorder (ARFID), and pica. EDNOS is no longer recognized in the Diagnostic and Statistical Manual of Mental Disorders (DSM-5) [19], but it is still used in ICD-10. Diagnostic information was obtained from the Danish National Patient Register [20, 21] and Danish Psychiatric Central Research Register [22]. These registers contain diagnoses from all inpatient contacts before 1995 and both in- and out-patient contacts from 1995 onwards. Information on sex, birth year, death, and emigration was obtained from the CRS [18].

**Table 1:** Diagnostic groups based on ICD-10 codes for cohort studies and The Lundbeck Foundation Initiative for Integrative Psychiatric Research (iPSYCH), the Anorexia Nervosa Genetics Initiative (ANGI), and the Eating Disorders Genetics Initiative (EDGI) case-cohort sample for attention-deficit/hyperactivity disorder (ADHD), autism spectrum disorder (ASD), anorexia nervosa (AN), bulimia nervosa (BN), and eating disorder not otherwise specified (EDNOS).

Co-occurrence and familial co-aggregation studies	ICD-10
ADHD	F90.x, F98.8
ADHD (narrow)	F90.0
ASD	F84.0, F84.1, F84.5, F84.8, F84.9
AN	F50.0, F50.1
BN	F50.2, F50.3
EDNOS	F50.8, F50.9
Any ED	F50.0, F50.1, F50.2, F50.3, F50.8, F50.9
Mood disorder	F30-F39
Anxiety disorder	F40-F48
Case definition iPSYCH, ANGI, and EDGI selection	
ADHD	F90.0
ASD	F84.0, F84.1, F84.5, F84.8, F84.9
AN	F50.0, F50.1

### Genetic data

Genotypes were generated from samples obtained from the Danish Neonatal Screening Biobank, which stores dried blood spots taken after birth from nearly all infants born in Denmark since 1981. The samples used in this study were originally genotyped for The Lundbeck Foundation Initiative for Integrative Psychiatric Research (iPSYCH) [23, 24], a large-scale genetic study nested within our study population, and Danish cohorts of the Anorexia Nervosa Genetics Initiative (ANGI) [25] and the Eating Disorders Genetics Initiative (EDGI) [26], which used the same sampling method for cases as iPSYCH. Possible batch effects were captured by a sample cohort variable. For this study, we included individuals with AN (*N*_cases_ = 7012; *N*_controls_ = 45,132), ADHD (*N*_cases_ = 28,183; *N*_controls_ = 44,149), and ASD (*N*_cases_ = 20,773; *N*_controls_ = 44,644) from the iPSYCH, ANGI, and EDGI cohorts as described in Table 1. None of these cohorts specifically sampled BN and EDNOS, and genetic analyses therefore focused on associations with AN only. Controls without the disorder of interest were drawn from a sub-cohort that included a random 2% subsample of the birth population. PGSs for AN [14], ADHD [11], and ASD [13] were calculated using LDpred2 [27] and GWAS summary statistics excluding Danish samples. PGSs were standardized by subtracting the mean and dividing by the standard deviation (SD) calculated using controls only.

### Statistical analysis

Analyses described in the following paragraphs were conducted using Stata software version 16.

#### Co-occurrence risks between ADHD or ASD and EDs

We first estimated hazard ratios (HRs) with 95% confidence intervals (CIs) for each ED following ADHD or ASD diagnosis using Cox proportional hazard models, adjusted for age as the underlying time scale, birth year as a categorical covariate, and stratified by the registered sex assigned at birth, except if an individual had their registered sex changed (henceforth referred to as sex) in the underlying hazard. ADHD and ASD were handled as time-varying exposures, meaning individuals were considered unexposed until receiving an ADHD or ASD diagnosis. The rate of an ED diagnosis in the exposed group was compared with a same-age unexposed group. Individuals were followed from their sixth birthday until death, emigration, ED diagnosis, or December 31, 2016, whichever came first. In the registers, ED diagnoses are occasionally registered before ADHD or ASD diagnosis. The analysis was consequently also conducted with EDs as time-varying exposures and ADHD or ASD as outcomes. In all Cox regression models, a cluster-robust sandwich estimator was used for standard errors (SEs) and CIs to account for dependencies between individuals with the same mother.

We performed sensitivity analyses to examine the reliability of our co-occurrence risks results. Analyses with mutually adjusted ADHD and ASD were conducted to assess whether an association between EDs and ADHD was explained by comorbidity with ASD and vice versa. In the iPSYCH cohort, a narrower definition of ADHD (F90.0) was used. Thus, a sensitivity analysis narrowing the definition of ADHD to individuals diagnosed with disturbance of activity and attention was performed.

#### Influence of mood and anxiety disorders on risk for ED following ADHD or ASD diagnosis

To assess the potential mediating effects of intermediate mood or anxiety disorders on the association between ADHD or ASD as exposures and EDs as outcomes, an analysis using mood or anxiety disorders as an additional time-varying covariate was performed. We performed four-way decomposition mediation analysis [28] with mood or anxiety disorders as potential time-dependent mediators between ADHD or ASD (as exposure, assumed to be present from start of follow-up at age 6) and EDs (as outcome) to estimate the direct effect of ADHD or ASD on ED risk, and the indirect effect mediated by intermediate mood or anxiety disorders. Only mood and anxiety disorders diagnosed prior to EDs were included, but they could be present from the start of follow-up.

#### Familial co-aggregation analysis

We identified all possible pairs of full siblings, half siblings, and cousins in the study population to study heritability in first-, second-, and third-degree relatives. The diagnostic status of each person was evaluated at the end of follow-up. Using logistic regression, we estimated odds ratios (ORs) with 95% CIs of EDs among individuals with each type of relative diagnosed with either ADHD or ASD compared to individuals with the same type of relative not diagnosed with that disorder. All logistic regression models were adjusted for both the index person’s and relative’s sex and birth year.

#### Cross-disorder polygenic association

We estimated the association between ADHD-PGS or ASD-PGS and the risk of AN diagnosis, and vice versa. ORs per SD increase in PGS and per quintile (with the lowest quintile as reference) were estimated in a weighted logistic regression model using the Kalbfleisch and Lawless estimator [29] with robust SEs (where weights are 1 for individuals with AN, ADHD or ASD diagnosis whereas weights for controls from the sub-cohort were calculated as the inverse probability of being selected for the sub-cohort [23]). All analyses were adjusted for sex, birth year, sample cohort variable, and population stratification using the first five genetic principal components [30, 31]. We were interested in obtaining estimates of the association between the ADHD-PGS or the ASD-PGS with AN diagnosis accounting for major depressive disorder (MDD) genetics because MDD frequently co-occur with AN, ADHD and ASD. Hence, we carried out sensitivity analysis in which we included MDD-PGS [32] as a covariate in the model. AN was split into AN without lifetime BN and AN with lifetime BN as proxy measures of AN subtype to examine whether ADHD or ASD PGS associations varied between these AN diagnostic subtypes (i.e., characterized by the presence/absence of binge eating and purging). The GWASs used to calculate PGSs were based on individuals with European ancestry. Thus, we performed a sensitivity analysis limiting our study population to individuals with European ancestry.

### Ethics and data approvals

The iPSYCH [23], ANGI [25], and EDGI [26] studies were approved by the Danish Scientific Ethics Committee, the Danish Health Data Authority, the Danish Data Protection Agency, and Danish Newborn Screening Biobank Steering Committee. The Danish Scientific Ethics Committee, in accordance with Danish legislation, has waived the need for informed consent in biomedical research based on existing biobanks. The study was approved by the Danish Data Protection Agency, and data access was approved by Statistics Denmark and the Danish Health Data Authority. All data were de-identified and not recognizable at an individual level.

## Results

The study population comprised 1,671,823 individuals (814,047 females and 857,776 males). Supplementary Fig. 1 shows the cumulative incidence of EDs, ADHD, and ASD by sex and age. The prevalence of any ED overall was 0.94%; individuals with either ADHD or ASD had a higher prevalence of any ED (2.01% and 2.57%, respectively) compared to individuals without ADHD or ASD (0.91% in both groups). Some case groups are overlapping as individuals could be diagnosed with multiple EDs and both ADHD and ASD. More information on the demographics of the Danish population can be found elsewhere [33, 34].

### Co-occurrence risk between ADHD or ASD and EDs

**Fig. 1:**
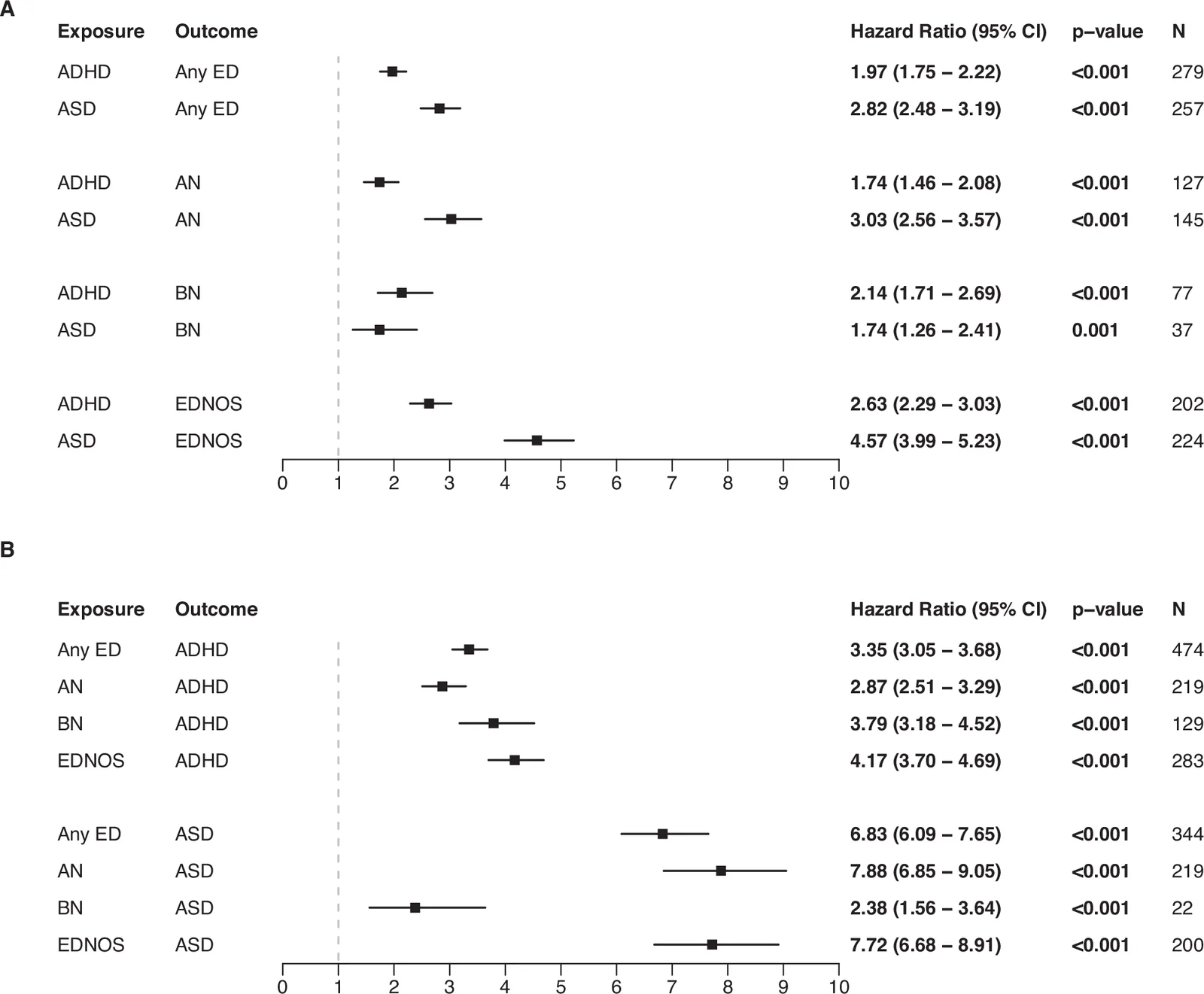
Hazard ratios for within-individual associations. **A** Association between prior ADHD or ASD and subsequent EDs. **B** Association between prior EDs and subsequent ADHD or ASD.

Increased risk for all EDs was observed in individuals first diagnosed with either ADHD or ASD (Fig. 1A). Further, individuals in each ED diagnostic group (i.e., Any ED, AN, BN, EDNOS) had an increased risk of a subsequent ADHD or ASD diagnosis (Fig. 1B). Adjusting for co-occurring ADHD and ASD in individuals only slightly attenuated associations with a later ED diagnosis (Supplementary Table 1). Individuals with co-occurring ADHD and ASD were thus included in both the ADHD and the ASD case groups. The time between first and second diagnoses was shorter among individuals diagnosed with an ED prior to ASD than those diagnosed with ASD first (Supplementary Table 2); this pattern was not observed between ADHD and EDs. Also, narrowing the definition of ADHD to individuals diagnosed with F90.0 did not change the results (Supplementary Table 3).

### Influence of mood and anxiety disorders on risk for ED following ADHD or ASD diagnosis

**Fig. 2:**
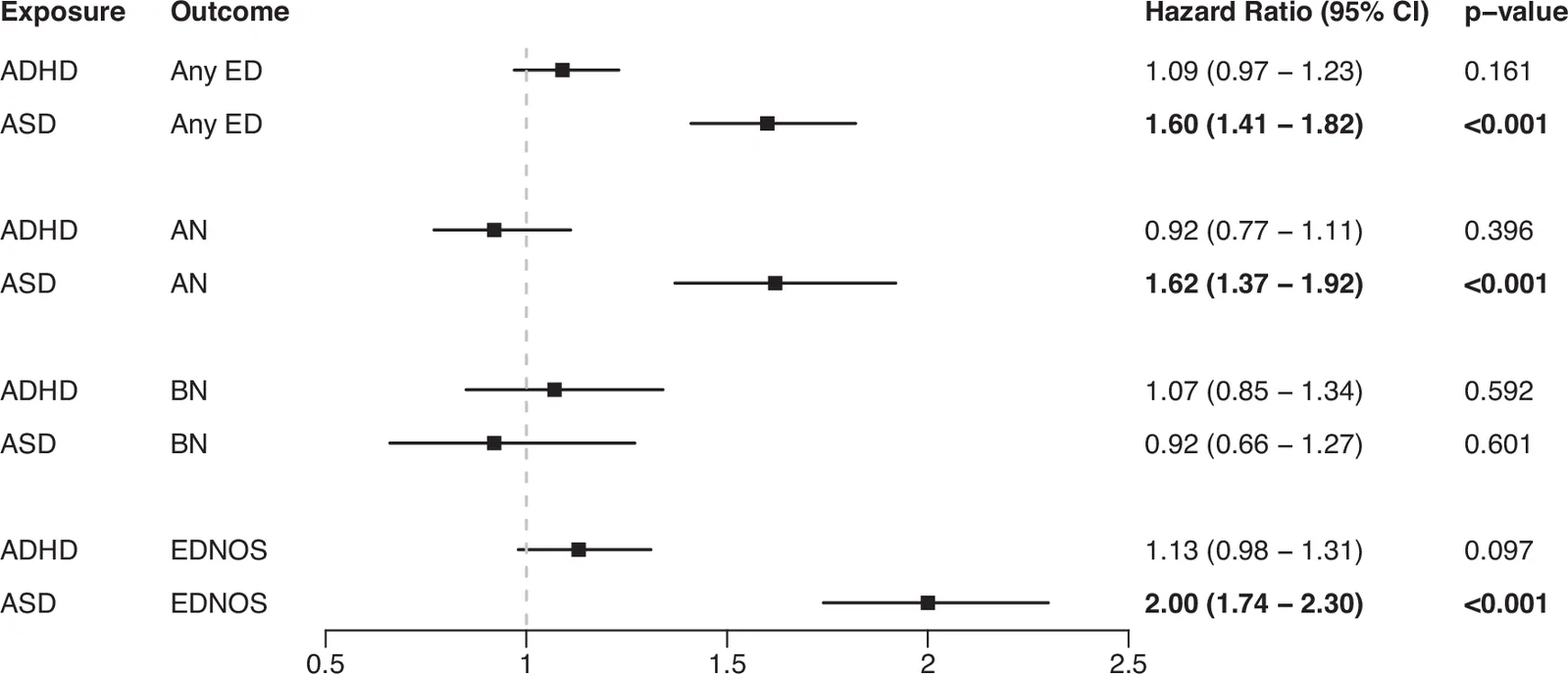
Hazard ratios for within-individual associations between prior ADHD or ASD and subsequent EDs, adjusted for presence of mood disorders and/or anxiety disorders. Influence of mood and anxiety disorders on risk for ED following ADHD or ASD diagnosis.

Adding mood or anxiety disorders as a covariate reduced all ED risks following ADHD or ASD diagnosis and led to non-significant associations between all EDs (including the ‘Any ED’ category) following ADHD as time-varying exposure, and between BN following ASD as time-varying exposures (Fig. 2).

**Fig. 3:**
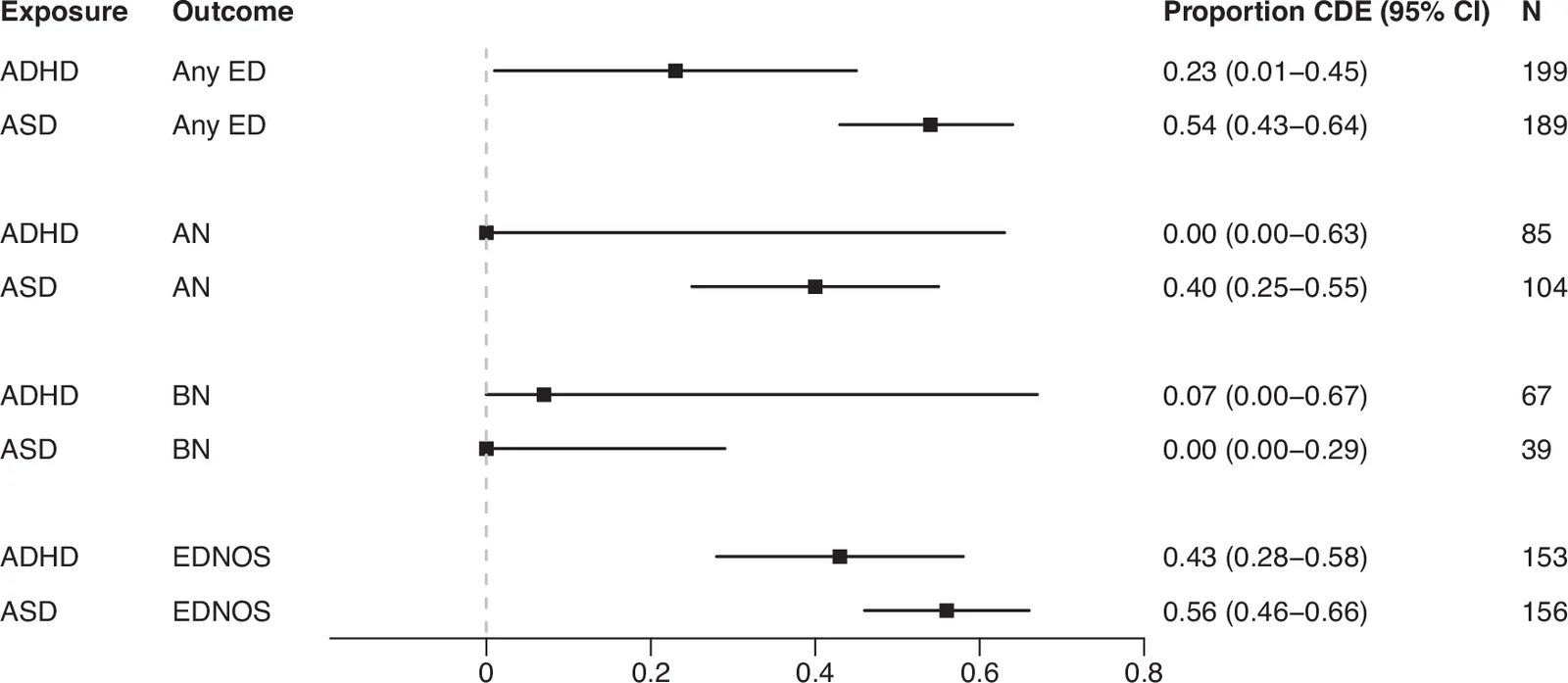
Proportion of association attributed to the controlled direct effect (CDE). Results from four-way decomposition mediation analysis with mood and/or anxiety disorders as mediator.

The mediation analysis showed that the estimated direct association between ADHD and AN or BN and between ASD and BN were small when mood or anxiety disorders were included as a mediator (Fig. 3). For associations between all other EDs (including the ‘Any ED’ category) and ADHD or ASD, the estimated direct effects ranged from 23% to 56% when mood or anxiety disorders were included as a mediator between ADHD or ASD diagnosis and later EDs indicating that intermediate mood and anxiety disorder potentially account for between 44% and 100% of the associations between ADHD or ASD and EDs.

### Familial co-aggregation

**Fig. 4:**
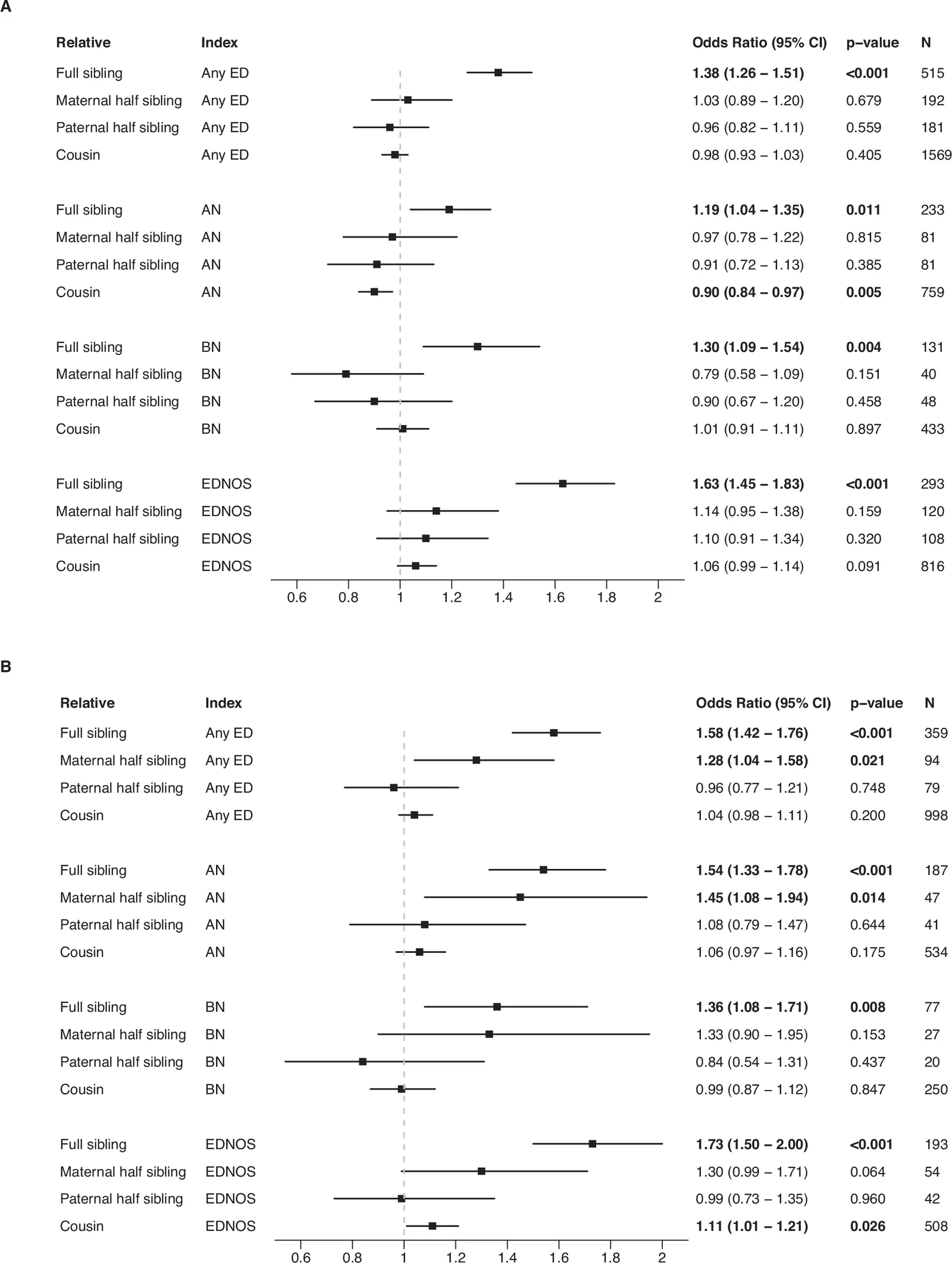
Hazard ratios for familial co-aggregation associations. **A** Association between relative ADHD and index person ED. **B** Association between relative ASD and index person ED.

The number of individuals linked to at least one of each relative type was: full sibling, 1,303,017 (77.94%); maternal half sibling, 249,130 (14.90%); paternal half sibling, 270,798 (16.20%); cousin 1,259,351 (75.33%). Individuals from the cohort with a full sibling diagnosed with ADHD or ASD had increased risks of all EDs compared to individuals with a sibling not diagnosed with that disorder (Fig. 4A, B, respectively). Also, individuals with a maternal half sibling with ASD had an increased risk of any ED and AN (Fig. 4B). A decreased AN risk for individuals with a cousin diagnosed with ADHD and an increased EDNOS risk for individuals with a cousin diagnosed with ASD was observed.

### Cross-disorder polygenic association

**Fig. 5:**
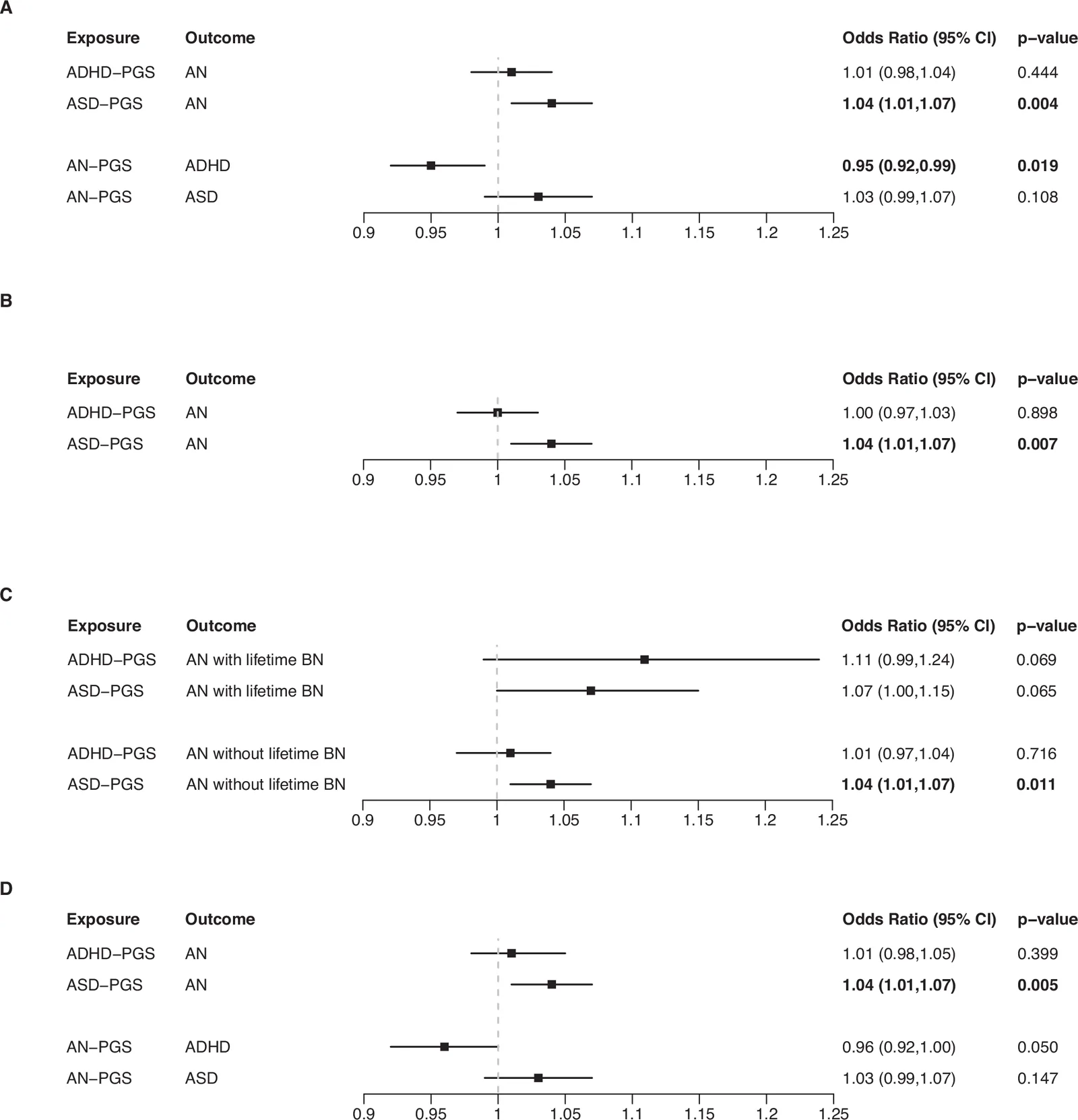
Odds ratios for within-individual associations between polygenic scores (PGSs) and diagnoses. **A** Associations between AN-PGS and diagnosis of ADHD or ASD, and vice versa. **B** Associations between ADHD-PGS or ASD-PGS and diagnosis of AN, adjusted for major depressive disorder PGS. **C** Associations between ADHD-PGS or ASD-PGS and diagnosis of AN with and without lifetime BN. **D** Associations between AN-PGS and diagnosis of ADHD or ASD, and vice versa. Cohort limited to individuals with European ancestry.

Individuals with AN had a higher ASD-PGS than controls, whereas we found no difference in ADHD-PGS between individuals with AN and controls (Fig. 5A; see Supplementary Fig. 2 for quintile plots). Furthermore, a lower AN-PGS was observed for ADHD cases compared to controls, but no differences in AN-PGS was observed for ASD cases compared with controls. Including MDD-PGS as a covariate did not alter the main results (Fig. 5B). Splitting individuals with AN into those with a lifetime BN diagnosis versus those without did not significantly change the association between ADHD-PGS and AN. The association between ASD-PGS and AN cases with lifetime BN diagnosis was not significant whereas the higher association between ASD-PGS and AN cases without lifetime BN diagnosis remained significant (Fig. 5C). Limiting the cohort to individuals of European ancestry also did not alter the results, except the association between AN-PGS and ADHD cases was no longer significant (Fig. 5D). Of note, the proportion of individuals with European ancestry was 93.82%, 91.99%, and 91.37% in the analyses for AN, ADHD or ASD as the outcome, respectively.

## Discussion

In this nationwide, population-based study, we found significant co-occurrence risk for EDs with either ADHD or ASD. Further, we report the novel finding that the risk of ADHD or ASD and later EDs is strongly mediated by intermediate mood or anxiety disorders. We also observed consistent cross-disorder risks for all combinations of EDs and ADHD or ASD among full siblings, indicating that familial factors may contribute to the co-occurrence risks. Increased risk of AN in individuals with a maternal half sibling with ASD indicates that genetic factors may contribute to the co-occurrence risk. This association between ASD and AN could potentially be explained by current measures of common genetic variation.

The overall prevalence of any ED in the study population is consistent with previous studies using Danish register data [33–35]. We found bidirectional associations between EDs and ADHD or ASD, although the strength of the associations varied by ED diagnosis. Regardless of which disorder was diagnosed first, there was an overall stronger association between ADHD and BN or EDNOS than between ADHD and AN. Similarly, we observed a stronger association between ASD and AN or EDNOS than between ASD and BN. These findings are consistent with a previous study [10] that reported a stronger association between ADHD and EDs other than AN, as well as a systematic review demonstrating that ASD is more common in individuals with AN than individuals with other EDs [2].

The association between EDNOS and ASD was stronger compared to associations between ASD and AN or BN. This could be explained in part by a lower age at EDNOS diagnosis among individuals with an ASD diagnosis, suggesting that some individuals with ASD likely had symptoms pertaining to feeding from infancy [36] that could have been classified as EDNOS. This finding is consistent with clinical studies reporting a high prevalence of ARFID among individuals with ASD [37], and higher co-occurrence between ASD and ARFID than between ASD and AN or BN [38]. This association has important clinical implications, as individuals with ARFID restrict food intake due to sensory aspects of food, a lack of interest in food and/or fear of negative consequences when eating or a combination of these symptoms, but typically restriction is not motivated by a fear of weight gain as seen in AN [39]. A Swedish twin study estimated the ARFID population prevalence to be 2.0% [40], which could be comparable in the Danish population. Although ARFID was formally recognized in DSM-5 in 2013, ARFID has no unique ICD-10 code in the Danish registers and is classified under EDNOS (F50.8-F50.9). It is thus not possible to distinguish ARFID in the Danish registers.

Although we observed strong associations between EDs as exposures and ADHD or ASD as the outcome, we urge caution when interpreting the order of diagnosis. Although ADHD and ASD are shown to be neurodevelopmental in origin and have an early-life onset, there is considerable variation in the age at diagnosis, with additional differences by sex (Supplementary Fig. 1). In females, there is a marked increase in the rate of new diagnoses of ADHD and ASD during early to mid-adolescence, whilst males are more commonly diagnosed during childhood [41]. As adolescent rates of EDs also increase among females [34], the associations between EDs and subsequent ADHD or ASD may reflect these age- and sex-specific diagnostic patterns, influenced by the effects of both social and biological changes in early adolescence. These could also be milder cases of ADHD/ASD that are detected later in life under more careful examination when the patients are evaluated for their ongoing ED. Low counts of individuals in some ED groups prevented sex-specific analysis.

A novel finding of this study is that most of the associations observed between ADHD or ASD as exposure and later ED were largely accounted for by the mediating effects of intermediate mood or anxiety disorders. That is, individuals with ADHD or ASD were more likely to develop a mood/anxiety disorder, which in turn increased their risk of developing an ED. Our results suggest that intermediate mood and anxiety disorders could account for 44% or more of subsequent ED diagnoses in people with ADHD or ASD. This is consistent with past research indicating that relationships between EDs and neurodevelopmental disorders can be mediated by mood- and stress-related factors including alexithymia, negative affect, and emotion dysregulation [42, 43]. Taken together, this association points to a potential intervention opportunity to mitigate ED development in individuals with ADHD or ASD by rapidly detecting and treating co-occurring mood or anxiety disorders.

Our results also showed that individuals with a full sibling with ADHD or ASD had increased risk for all EDs. Also, individuals with a maternal half sibling with ASD had an increased risk of any ED and AN, which supports the presence of a shared genetic influence between ASD and AN [13, 14]. As maternal half siblings are more likely to share a home in childhood this could also indicate that shared environmental factors contribute to the association between EDs and having maternal half siblings with ASD but not paternal half siblings with ASD. Although no statistically significant associations were observed between other relatives diagnosed with ADHD and increased ED risk in the index person, our results do not contradict findings in the Swedish population study showing a positive correlation between individuals who had a maternal half sibling with ADHD and increased risk of EDs other than AN [10]. The wide confidence intervals should warrant caution when interpreting these results, which could suggest that our analyses of half siblings may be underpowered compared to the Swedish study.

We found no associations between ADHD-PGS and AN diagnosis, but a small negative correlation between AN-PGS and ADHD diagnosis, indicating that the positive diagnostic correlations do not appear to be due to shared genetic influence, at least as measured by current PGSs. Studies of genetic correlations between AN and ADHD have found negative correlation [11], positive correlation [15], or no correlation [12, 14, 44]. The positive association between ASD-PGS and AN diagnosis supports the hypothesis that AN and ASD may have a shared genetic architecture. Our results align with prior studies showing small but positive genetic correlations between AN and ASD [13, 14], although a negative correlation has also been reported [15]. The small genetic correlation between AN and ADHD or ASD could be due to insufficient sample size (both in the discovery GWASs and our study) and PGSs only capturing common genetic variation at single nucleotide polymorphism level. Of note, a recent examination of the genetic architecture of major psychiatric disorders revealed low genetic correlation between AN and ADHD or ASD [45], suggesting that AN is not a neurodevelopmental disorder like ADHD and ASD.

We were unable to examine AN restricting versus AN binge-eating/purging subtypes separately since subtype data are not available in Danish registers. Examining individuals with AN with versus without a lifetime BN diagnosis as a proxy measure for AN subtypes yielded slightly higher estimates for associations between AN with lifetime BN and ADHD-PGS, suggesting that ADHD could be more strongly associated with the AN binge-eating/purging subtype than the restricting subtype, as suggested in previous studies [7, 46]. Furthermore, the results suggest that ASD could be more strongly associated with the AN restricting subtype than the binge-eating/purging because the association between AN with lifetime BN diagnosis and ASD-PGS was not significant whereas the association between AN without lifetime BN diagnosis stayed significant.

### Strengths and limitations

This study used nationwide registers that only capture diagnoses reported by specialists in hospital settings. Therefore, individuals diagnosed in primary care with subthreshold disorders, who are not in contact with hospital-based specialists, or individuals that do not seek help were not captured. The true prevalence of EDs is expected to be higher in the Danish population as seen in the Finnish population where only 32% of those diagnosed in an interview self-reported to have ever been diagnosed by healthcare professionals. For AN, 57% of those diagnosed by the interview reported to have an earlier diagnose from a healthcare professional [47]. Danish registry data on psychiatric diagnoses generally have good validity with high positive predictive values [48, 49]. ED diagnoses in the Danish registers have not been validated, although EDs from highly similar Swedish register data show good validity [50]. The registers only provide data on the date of first diagnosis, not the actual time of onset. Some degree of detection bias is probable, and the very high incidence of ASD diagnosis subsequent to ED diagnosis may in part be due to this detection bias or reflect the impact of starvation on cognitive and social functioning in females with undiagnosed ASD.

No large GWASs for EDs other than AN are currently available, therefore not allowing the inclusion of PGSs for BN and EDNOS. Further, current PGSs are underpowered and capture only a fraction of the estimated heritability due to existing GWAS sample sizes. Of note, Danish contributions constitute a large proportion of available GWAS training samples for AN, ADHD, and ASD. Therefore, exclusion of Danish samples from the training samples further limits the power of PGSs based on summary statistics. Replication of the PGS analysis will be necessary as larger GWASs and PGSs with increased power become available.

## Conclusion

We observed a strong and consistent diagnostic overlap between EDs and ADHD or ASD, which strongly points to the role of genetics in the association between ASD-PGS and AN. First, in analyses serving as a proxy for genetic liability by PGSs, the ASD-PGS showed a positive association with AN and the AN-PGS showed a negative association with ADHD. Second, we observed significant associations between any ED or AN and ASD for maternal half siblings in family analysis, as well as co-aggregation in full siblings for all EDs and ADHD or ASD. Overall, these results highlight considerable gaps in our knowledge of the neurodevelopmental mechanisms linking EDs to ADHD and ASD. Nevertheless, our mediation analysis results underscore that the associations between EDs and ADHD or ASD appear to be strongly mediated by intermediate mood or anxiety disorders. These findings highlight opportunities to mitigate the development of EDs among individuals with ADHD or ASD by screening and treating emergent mood and anxiety disorders as early as possible.

## Supplementary information

Supplementary material

## Data Availability

The register data that support the findings of this study are not publicly available but can be applied for from Statistics Denmark. The summary statistics used in this publication are available from the Psychiatric Genomics Consortium, https://pgc.unc.edu/ and 23andMe, https://www.23andme.com/en-eu/. For access to all relevant iPSYCH, ANGI, and EDGI data, please find contact information at https://ipsych.dk/.

## Code availability

All analyses were performed on a secure server and code will not be made publicly available.
