# Supplementary material for: The role of co-occurring conditions and genetics in the associations of eating disorders with attention-deficit/hyperactivity disorder and autism spectrum disorder
Source: Mol Psychiatry. 2024 Nov 14;30(5):2127–36. doi: 10.1038/s41380-024-02825-w (PMC12014370; doi:10.1038/s41380-024-02825-w)

Supplementary information

**Supplementary Table 1.** Co-occurrence risk between prior, mutually adjusted ADHD and ASD and later ED


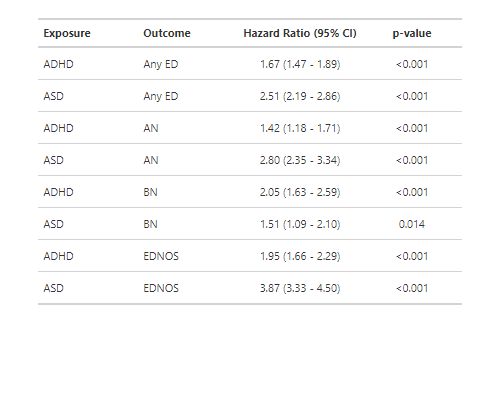


**Supplementary Table 2.** Time between first and second diagnoses in individuals diagnosed with both an ED and ADHD or ASD, depending on order of diagnoses. Median and interquartile range (IQR), rounded to nearest tens to comply with Danish data protection rules.


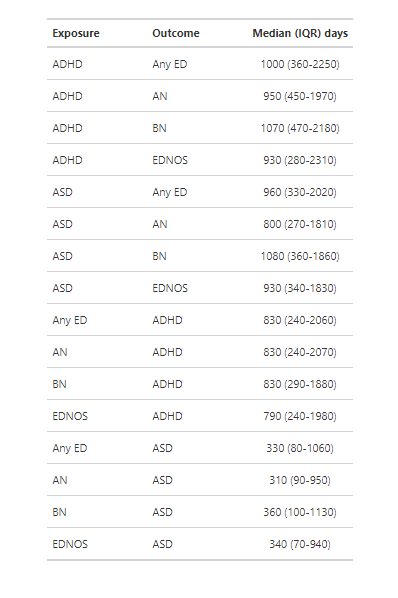


**Supplementary Table 3.** Associations between EDs and ADHD (narrow definition - diagnoses of F90.0 only)


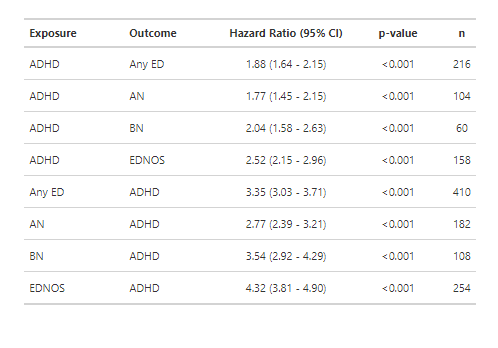


**Supplementary Figure 1.** Cumulative incidence of included disorders by age and sex.


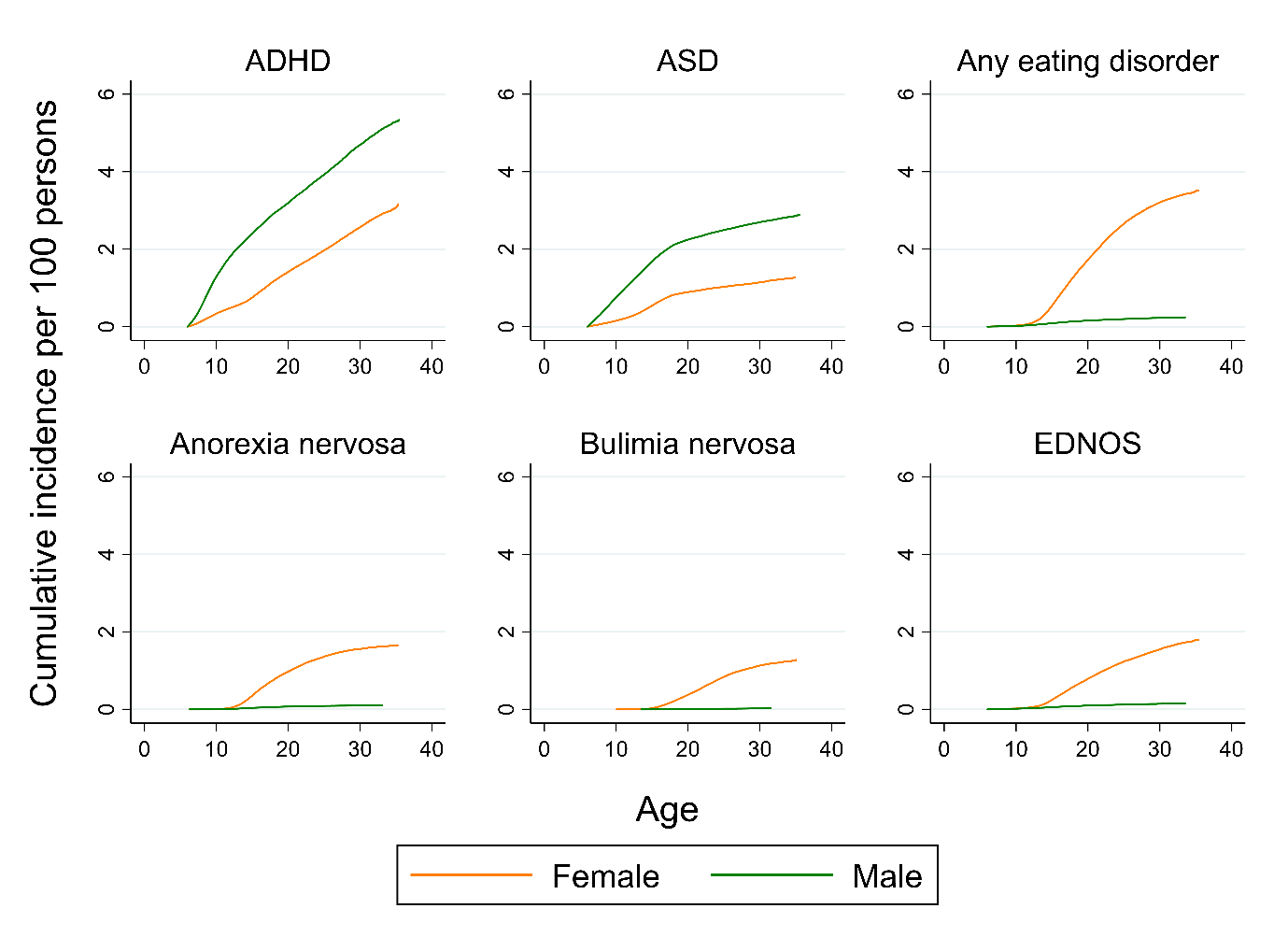


**Supplementary Figure 2.** A) Hazard ratios for within-individual associations between quintiles of ADHD-PGS or ASD-PGS and diagnosis of AN, with first quintile (i.e., AN cases who are in the bottom 20% for ADHD or ASD PGS) as referent. B) Hazard ratios for within-individual associations between quintiles of AN-PGS and diagnosis of ADHD or ASD, with first quintile (i.e., ADHD or ASD cases who are in the bottom 20% for AN PGS) as referent.


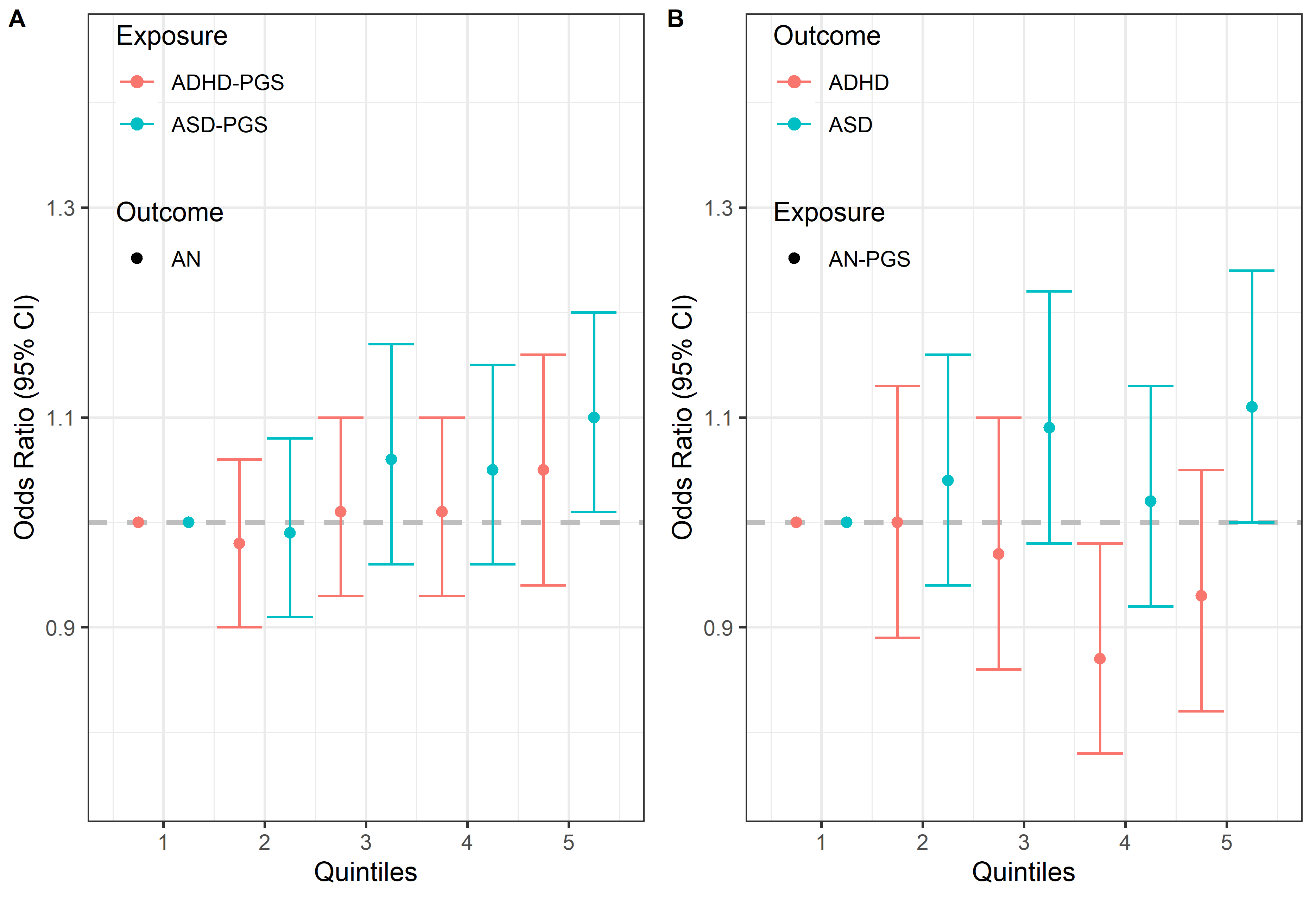

Supplement: Supplementary file 1 — Supplementary material [file 41380_2024_2825_MOESM1_ESM.docx]
